# An in vitro evaluation of standard rotational thromboelastography in monitoring of effects of recombinant factor VIIa on coagulopathy induced by hydroxy ethyl starch

**DOI:** 10.1186/1471-2326-5-3

**Published:** 2005-02-15

**Authors:** Martin Engström, Peter Reinstrup, Ulf Schött

**Affiliations:** 1 Department of Anaesthesia and Intensive Care, Lund University Hospital, Sweden; 2 Department of Anaesthesia and Intensive Care, Halmstad County Hospital, Sweden

## Abstract

**Background:**

Rotational thromboelastography (ROTEG) has been proposed as a monitoring tool that can be used to monitor treatment of hemophilia with recombinant factor VIIa (rFVIIa). In these studies special non-standard reagents were used as activators of the coagulation. The aim of this study was to evaluate if standard ROTEG analysis could be used for monitoring of effects of recombinant factor VIIa (rFVIIa) on Hydroxy Ethyl Starch-induced dilutional coagulopathy.

**Methods:**

The study was performed in vitro on healthy volunteers. Prothrombin time (PT) and ROTEG analysis were performed after dilution with 33% hydroxy ethyl starch and also after addition of rFVIIa to the diluted blood.

**Results:**

PT was impaired with INR changing from 0.9 before dilution to 1.2 after dilution while addition of rFVIIa to diluted blood lead to an overcorrection of the PT to an International Normalized Ratio (INR) value of 0.6 (p = 0.01). ROTEG activated with the contact activator ellagic acid was impaired by hemodilution (p = 0.01) while addition of rFVIIa had no further effects. ROTEG activated with tissue factor (TF) was also impaired by hemodilution (p = 0.01) while addition of rFVIIa lead to further impairment of the coagulation (p = 0.01).

**Conclusions:**

The parameters affected in the ROTEG analysis were Clot Formation Time and Amplitude after 15 minutes while the Clotting Time was unaffected. We believe these effects to be due to methodological problems when using standard activators of the coagulation in the ROTEG analysis in combination with rFVIIa.

## Background

Patients undergoing massive hemorrhage experience dilutional coagulopathy with crystalloid and/or colloid resuscitation. If hemorrhage progresses, packed red cells (RBC) are transfused together with crystalloids and/or colloids. Regarding the coagulation this is not optimal, and the patients often develop a dilutional coagulopathy, sometimes worsened by hypothermia. In addition a coagulopathy caused by the administration of dextrane or hydroxy ethyl starch (HES) may be induced. The common approach to this is transfusion of fresh frozen plasma (FFP) and platelets, but bleeding might continue, with often fatal outcome. Prophylactic use of fresh frozen plasma (FFP) or platelet transfusion is not proven beneficial to prevent hemorrhage in massively transfused patients[1]. Hemorrhage, complicated by the development of coagulopathy, is therefore still the major cause of death in trauma patients arriving alive in the hospital.[2,3].

A novel approach to treat these patients is the use of recombinant factor VIIa (rFVIIa) to improve the coagulation.[4]. Recently there have been several case publications of successful treatment of coagulopathic trauma patients and surgical patients. [5-7].

**Figure 1:**
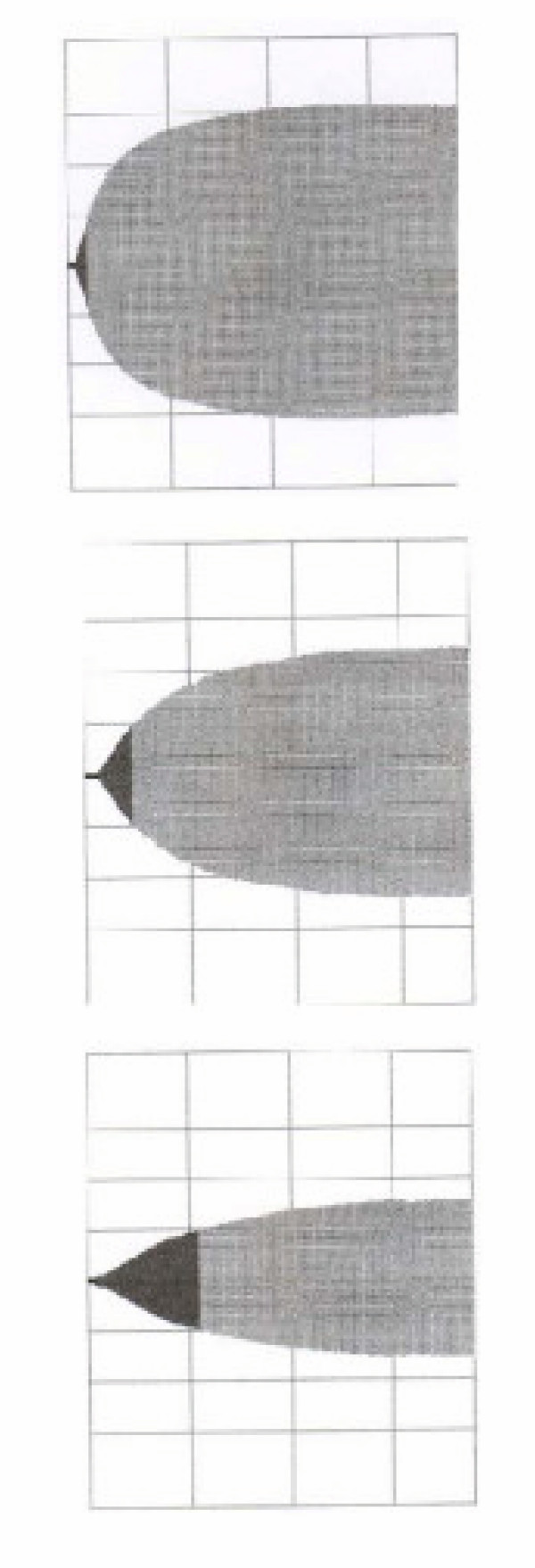
The figure shows the 3 representative tracings of the EXTEG analysis from one of the participants in the study. Above the normal tracing with a short CT and CFT is shown. It can be seen that the clot strength is rapidly increasing after initiation of the clotting. In the middle the tracing after hemodilution with HES is found. It can be seen that the CFT is prolonged and that the strength of the clot is increasing slower. Below the tracing after hemodilution and addition of rFVIIa is found. The clotting is then severely impaired, the clot strength is increasing slower and the maximum strength is also severely impaired.

**Figure 2:**
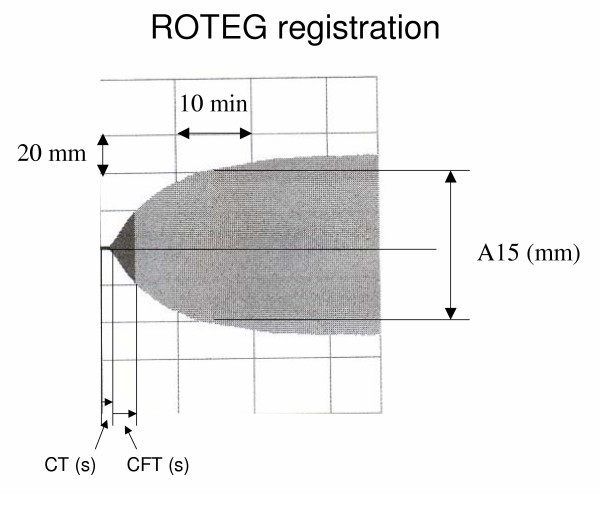
The figure shows the result of a ROTEG analysis. Time is represented on the X axis and clot strength on the Y axis. The clot strength is arbitrarily measured in mm where maximum clot strength is 100 mm. The Clotting Time (CT) is the time from initiation of the analysis until the clot strength is 2 mm. The Clot Formation Time (CFT) is the time from clot strength 2 mm until clot strength 20 mm. A15 is the clot strength at 15 minutes.

Dilutional coagulopathy can be detected by the use of thromboelastographic measurements [8-11]. Rotational thromboelastography (ROTEG) is a recent development of thromboelastography. ROTEG gives a viscoelastic measurement of clot strength in whole blood. It is presented as a graph representing clot strength during the build-up of a clot (figures 1 and 2). From the graph several variables describing different parts of the coagulation process are derived and measured numerically. It thus gives a more comprehensive picture of the coagulation than standard tests, but is on the other hand less validated and standardised than the more common coagulation tests. An advantage of the method is that it can be used as a point-of-care analysis. One condition to be fulfilled if it should be used as a point-of-care method is that the commercially available kits can be used for analysis and that special preparations and/or dilutions should not be necessary, as that would require the skills and competencies of a full laboratory.

Previous studies have shown that hemodilution with HES impairs the coagulation already at a dilution of 33% while crystalloid hemodilution does not appear to give a readily detectable impairment of the coagulation until the level of hemodilution reaches 50% [10,12]. In this study we have investigated if ROTEG can be used with the commercially available kits to monitor effects of rFVIIa and if rFVIIa is able to improve the coagulopathy caused by hemodilution with HES.

## Methods

The local ethics committee of the University of Lund approved this study on healthy volunteers. Volunteers were not allowed to take any medication 14 days prior to the study. Informed consent was obtained from the participants and a total of eight were recruited. All participants had an indwelling intravenous catheter placed into the brachiocephalic vein, from which the blood samples were drawn with sterile disposable 5-ml syringes (Luer; Codan Medical Aps, Rödby, Denmark). A first 5-ml blood sample was discarded before every blood sample for the experiment described below. No tourniquet was used on the arm when samples were drawn.

Dilution of the blood samples was performed with HES 130/0.4 (Voluven^®^, Fresenius Co., Bad Homburg, Germany), the HES preparation found to cause the least pronounced coagulopathy after hemodilution [12]. Three different preparations of blood were examined. The first preparation contained 5 ml of undiluted blood (normal). The second preparation contained 3.3 ml of blood and 1.7 ml of HES thereby achieving a 33% dilution (dilution). The third preparation contained 3.3 ml of blood, 1.7 ml of HES and 50μl of rFVIIa at the concentration 0.12 μg/μl (dilution + rFVIIa). The latter concentration of rFVIIa was equivalent to the concentration achieved when the dose 90 μg/kg body weight is administered in vivo. 90 μg/kg is the recommended dose in hemophilia and well within the range suggested for treatment of acute hemorrhage in non hemophilia patients.[7,13]. The reason for the chosen dilution (33%) was that it is a clinically relevant dilution that is readily achieved during resuscitation of a patient. Before initiating this study we have also tested 50% dilution in a single person and the results were similar to the results with 33% dilution, but more pronounced. We then decided to study the 33% dilution systematically. Further on, this dilution did not induce unphysiologic changes in Ca or pH as tested in a pilot volunteer. The dilution was performed in a polypropylene test tube and the tube was gently turned to mix the blood with the added HES and rFVIIa. The HES was warmed in a heating block (Grant Instrumentation Ltd, Cambridge, UK) to 37°C prior to hemodilution in order to avoid hypothermia as a confounding factor after dilution.

The tests performed on the different preparations were hemoglobin concentration (Hb), Prothrombin time (PT) and ROTEG analysis. All tests were performed at normal body temperature (37°C). For the Hb measurements a Hemocue (HemoCue Co., Ängelholm, Sweden) was used. PT measurements were performed with a Rapidpoint Coag Analyzer (Bayer AB Diagnostics, Gothenburg, Sweden) with PT-ONE test cards. The ROTEG analyses were performed on a Rotational Thromboelastograph (ROTEG, Pentapharm, Munich, Germany) and the samples were analysed 120 seconds after the blood was drawn from the intravenous catheter. Both INTEG and EXTEG analyses were performed according to standard procedure recommended by the manufacturer. In INTEG analysis the coagulation is initiated with the addition of 20 μl of the contact activator ellagic acid (Pentapharm, Munich, Germany) to 320 μl of blood pipetted from the test tube to a reaction cup used in the ROTEG. In EXTEG analysis the coagulation is activated by the addition of 20 μl of a preparation containing tissue factor (TF) (Pentapharm, Munich, Germany) to 320 μl of blood pipetted from the test tube to the reaction cup. TF activates the coagulation through binding to Factor VIIa and this is believed to be the important interaction when in vivo coagulation occurs. The parameters obtained from the ROTEG analysis were Clotting Time (CT) reflecting the initiation of the coagulation, Clot Formation Time (CFT) reflecting the rate of clot formation once the formation is initiated and A15 describing the strength of the clot 15 minutes after initiation of the coagulation (figure 2).

Statistical analysis was performed with initial Kruskal-Wallis test and Wilcoxon's paired test was used when the Kruskal-Wallis test indicated a significant difference. All values are given as median (range). A p value of < 0.05 was considered statistically significant.

## Results

The insertion of venous catheters and the blood sampling were performed uneventfully. The Hb values decreased as an expected sign of dilution (table 1). PT values increased in the dilution group compared to normal and decreased to below normal in the dilution + rFVIIa group (table 1).

**Table 1:** Effects of dilution and addition of rFVIIa on Hb and PT values. A lowering of Hb and an increase in the PT were seen as signs of dilution while the addition of rFVIIa lead to a decrease of the PT (n = 8).

	Normal	Dilution	Dilution + rFVIIa
Hb (g/l)	136 (127–147)	88 (81–99)*	88 (81–99)*
PT (INR)	0.9 (0.7–1.2)	1.2 (0.9–1.3)^§^	0.6 (0.5–0.7)*^¶^

* p = 0.01 compared to normal. ^¶^p = 0.01 compared to dilution. ^§^p = 0.02 compared to normal.

In neither INTEG nor EXTEG analysis we found any change in the CT between normal and dilution groups, while CFT and A15 were impaired in the dilution group (tables 2 and 3). There were no differences between the dilution and the dilution + rFVIIa groups when analysed with the INTEG analysis (table 2). However, when rFVIIa was added to the dilution a prolongation of the CFT with > 200% and an impairment of the A15 with 40% were found (table 3 and figure 1).

**Table 2:** Coagulation variables as assessed by INTEG. Obvious signs of dilution are found in the CFT and the A15, while the addition of rFVIIa to the diluted blood does not affect the coagulation parameters (n = 8).

	Normal	Dilution	Dilution + rFVIIa
CT (s)	93.5 (82–104)	108.5 (87–136)	97.5 (68–118)
CFT (s)	85.5 (59–111)	216 (158–310)*	190.5 (167–368)*
A15 (mm)	56 (52–61)	43 (36–47)*	43.5 (33–50)*

* p = 0.01 compared to normal.

**Table 3:** Coagulation variables as assessed by EXTEG. Obvious signs of dilution are found in the CFT and the A15. The addition of rFVIIa to the diluted blood leads to a prolongation of the CFT and the A15 (n = 8).

	Normal	Dilution	Dilution + rFVIIa
CT (s)	51.5 (30–69)	63 (41–81)	58.5 (37–99)
CFT (s)	91 (67–105)	227 (171–332)*	558.5 (308–998)*^¶^
A15 (mm)	57 (53–63)	42 (33–49)*	25.5 (19–37)*^¶^

* p = 0.01 compared to normal. ^¶^p = 0.01 compared to dilution.

## Discussion

Treatment of dilutional coagulopathy is challenging and the primary monitoring tools are measurement of PT, activated partial thromboplastin time (APTT), platelet count and fibrinogen [14-16]. These tests provide us with information regarding the activation of the coagulation process and about the absolute number of platelets. However, they do not give us information regarding the dynamic properties of blood clotting and the rate at which the clot is formed once the clotting is initiated. New monitoring methods are needed and ROTEG is a monitoring tool that could potentially be of value in these situations. To this end, it has been suggested that treatment of haemophilia patients and liver transplant patients with rFVIIa can be monitored with ROTEG where a shortening of the CT and CFT has been found in case series. [17-19].

In our study we found that hemodilution in vitro with HES lead to augmentation of the PT and the addition of rFVIIa results in a prompt decrease of the PT. This is in line with previous studies, which have shown that dilution with HES lead to readily detectable changes in the coagulation system[9,12,20]. Former studies have also shown a decrease or a normalisation of the PT after administration of rFVIIa[21,22]. In this study we found an overcorrection of the PT to values below normal range.

When analysing the ROTEG parameters we found that the 33% dilution with HES resulted in a prolongation of CFT values and impairment in A15 values in accordance with previous studies[9,11,12,20]. The CT was not significantly prolonged even though there was a trend towards a prolongation of the CT in both INTEG and EXTEG. After rFVIIa had been added to the diluted blood, coagulation variables remained unchanged when assessed with INTEG, but were markedly affected when assessed with EXTEG. CFT and A15 reflect the dynamic interplay between platelets and fibrin polymerisation, both being disturbed by HES hemodilution as can be seen in table 2 and 3. Addition of rFVIIa *in vitro*, worsening both these parameters according to EXTEG analysis, suggested that platelets or fibrinogen became dysfunctional in contrast to the clinical effect of administration of rFVIIa, where rFVIIa has been found successful in case stories of bleeding patients [4,7,13,23], even though these cases may not have been bleeding due to a HES induced coagulopathy.

The lack of effect in the INTEG analysis when adding rFVIIa to the diluted blood is most likely due to the fact that a contact activator is used to activate the coagulation in the INTEG and therefore insensitive to rFVIIa as rFVIIa initiates coagulation through interaction with TF.

The impairment of CFT and A15 in the EXTEG analysis after addition of rFVIIa to the diluted blood is harder to explain. We expected addition of rFVIIa to the diluted blood to result in an improvement of the TEG parameters measured in the EXTEG analysis. This was expected partly because TF is used as an activator of the coagulation in the EXTEG analysis and the first step in the initiation of the coagulation system is the interaction between TF and FVIIa. [17,18,24]. In our study it is likely that the coagulopathy induced was at least to some extent caused by a platelet dysfunction caused by the colloid hemodilution. As bleeding caused by Glanzmann's thrombastenia and other thrombocytopathias is frequently and successfully treated with rFVIIa, is a reason why rFVIIa potentially could be effective in the treatment of this HES-induced coagulopathy[25-28].

The previously reported improvements of coagulation in hemophilia and liver transplant patients as evaluated with ROTEG after administration of rFVIIa also lead us to believe that ROTEG parameters would be improved after addition of rFVIIa to the diluted blood[17,18,24]. It is however important that these studies were performed on hemophilia patients suffering from a severe deficiency of factor VIII or IX and on liver transplant patients suffering from a very complex coagulopathy. It also seems important to dilute TF extensively to detect the effects of rFVIIa on ROTEG. Dilutions of TF up to 1:17000 have been performed by the Ingerslev group in Denmark [18,24]. These dilutions are, however, not made with commercially available reagents that are ready to use immediately and therefore not suitable for use outside research laboratories.

## Conclusion

In conclusion we found that 33% dilution of blood with HES 130/0.4 lead to impairment of the coagulation when evaluated with ROTEG or PT. Addition of rFVIIa lead to overcorrection of the prolonged PT. ROTEG analysis revealed that INTEG analysis was insensitive to effects of addition of rFVIIa and that EXTEG analysis was dramatically impaired by the addition of rFVIIa. It may well be that rFVIIa is not effective in improving the coagulopathy induced by HES hemodilution, but the further impairment of the coagulation seen in the EXTEG analysis is likely due to methodological problems. These problems make the commercially available EXTEG analyses inappropriate for monitoring of rFVIIa effects under circumstances of HES dilution.

## Competing interests

The author(s) declare that they have no competing interests.

## Authors contributions

ME contributed to the design of the study, performed the analyses and drafted the manuscript. PR contributed to the design of the study, to data interpretation and to preparing the manuscript. US contributed to the design of the study, performed the analyses and participated in manuscript preparation. All authors have read and approved the final manuscript.

## Pre-publication history

The pre-publication history for this paper can be accessed here:


